# Marijuana use and health behaviors in a US clinic sample of patients with sickle cell disease

**DOI:** 10.1371/journal.pone.0235192

**Published:** 2020-07-14

**Authors:** J. Deanna Wilson, Lydia H. Pecker, Sophie Lanzkron, Shawn M. Bediako, Dingfen Han, Mary Catherine Beach

**Affiliations:** 1 Department of Medicine, Division of General Internal Medicine, University of Pittsburgh, Pittsburgh, PA, United States of America; 2 Department of Pediatrics, Division of Adolescent and Young Adult Medicine, University of Pittsburgh, Pittsburgh, PA, United States of America; 3 Department of Pediatrics, Division of Pediatric Hematology, Johns Hopkins University School of Medicine, Baltimore, MD, United States of America; 4 Department of Medicine, Division of Hematology, Johns Hopkins University School of Medicine, Baltimore, MD, United States of America; 5 Department of Psychology, University of Maryland, Baltimore County, Baltimore, MD, United States of America; 6 Department of Medicine, Division of General Internal Medicine, Johns Hopkins University School of Medicine, Baltimore, MD, United States of America; University of Alabama at Birmingham, UNITED STATES

## Abstract

**Introduction:**

As marijuana use becomes more common, it is essential clinicians understand the relationship between marijuana use and health behaviors.

**Methods:**

Using a retrospective cohort of adolescents and adults with sickle cell disease (SCD) stratified into a young (<25 years) and older cohort (> = 25 years), we conducted multiple linear regression examining relationship of marijuana use (independent variable) on each dependent variable (SCD self-management score and pain management).

**Results:**

Among young cohort, 16.9% used marijuana compared to 21.8% of older cohort. The younger cohort reporting marijuana use had lower mean self-care scores (β = -2.74;p = 0.009) and were more likely to have admissions to the hospital for pain (β = 0.87;p = 0.047) compared to non-users. In contrast, the older cohort reporting marijuana use had more days treating pain at home (β = 0.44;p = 0.035).

**Conclusions:**

Only a minority of patients with SCD reported lifetime marijuana use. Among those reporting marijuana use, there were different associations with self-care and health-related behaviors by age. The older cohort who endorsed marijuana use reported more days of treating pain at home, although this did not translate into increased acute care visits for pain crisis. Among youth, endorsing marijuana use was associated with worse SCD self-care.

## Introduction

Individuals with sickle cell disease (SCD) suffer from recurrent episodes of acute pain and high rates of chronic pain [1]. Evidence suggests the use of marijuana to relieve pain and ancillary psychological symptoms may be common for a third to half of all SCD patients [2–7]. Some reports suggest SCD pain may be ameliorated by cannabis use [2–4], however, other studies identify potential harms, including an association with increased hospitalizations for pain in patients using cannabis although it is not clear that this is causal [5]. As clinicians become increasingly focused on evaluating the appropriateness of medical marijuana [6], more information about current marijuana use in patients with SCD is needed to inform approaches to counseling.

Our objective was to examine prevalence of marijuana use by age and if marijuana use was associated with SCD self-care behaviors and pain management. Evolving science has shown that the frontal lobes, and associated executive functioning, of adolescents and young adults continue to develop until age 25 [8]. Thus, adolescents and young adults are developmentally immature and place increased salience on peers and sensation-seeking behaviors and less on weighing substance-use related consequences than older adults [8]. Substance use occurring during this developmental period is often age-related experimentation and is statistically normative [9], although that does not preclude young people with chronic diseases also using to self-medicate pain or other symptoms.

Based on the growing recognition that emerging adults (up to 25 years of age) are an important group with unique drivers for substance use, we a priori stratified analyses to look at the effect of substance use in each of two age categories: adolescents and young adults less than 25 years of age and older adults greater than or equal to 25 years of age. We hypothesized there would be effect modification with a history of marijuana use associated with different self-care behaviors by age. We hypothesized older adults may use marijuana to manage pain (and thus marijuana use would be associated with positive self-care behaviors and higher self-care scores), while younger adults may use for pain, but are also more likely to use marijuana as part of normative experimentation, and thus marijuana use in the younger age cohort would not have associations to self-care.

## Materials and methods

This is a secondary data analysis from the Improving Patient Outcomes through Respect and Trust (IMPORT) Study, an observational cohort study using a convenience sample that received Institutional Review Board approval from Johns Hopkins Medical Institutions and Howard University [10,11]. Participants were recruited in the waiting rooms of outpatient adult and pediatric hematology clinics throughout 2010 and 2011, had to be 15 years of age or older at time of enrollment, diagnosed with a sickle cell hemoglobinopathy, report no plans to relocate in three years, and report willingness to adhere to study procedures. Patients completed a 45-minute computer-assisted audio self-interview and were reimbursed $50 for their time. We analyzed a subset of data collected at baseline asking about retrospective use of marijuana, self-care and pain-management behaviors (Included as supporting information, S1 File). Medical marijuana was not legally available during the survey period in Maryland or Washington, DC.

### Independent variable

#### Marijuana use

Participants were asked about any use of marijuana/cannabis in the past 30 days and also the number of years they used [12]. We dichotomized the sample into any marijuana use, which was coded as “lifetime use” and those who denied any use who and were coded “no use” for our analyses.

### Dependent variables

#### SCD self-care

Participants indicated how often (no reference time scale given as part of the question) they completed SCD-related self-care behaviors using a six question, five-point Likert scale from 0 (never) to 4 (very often) [13]. Sample questions included: “How often do you keep your clinic appointments?” “How often do you take medications as prescribed?” We totaled the score for each question and used the total score for analysis.

#### SCD pain management

Individuals were asked (1) how many times during a typical month they treated pain at home with possible response groupings from the original IMPORT study of 0, 1–5, 6–10, 11–20, >20; (2) how many times they were admitted to a hospital in the last 12 months with categories of 0, 1–2, 3–5, 6–10 or >10; and (3) how many times in the last 12 months they went to the ER or infusion center for a pain crisis, with response groupings of none, 1–2, 3–5, 6–10, >10.

### Statistical analyses

All analyses were conducted using the Stata 15.0 statistical software package (Statacorp v.15.0). Because of a priori hypotheses of effect modification by age, we stratified the sample by age into less than 25 and greater than or equal to 25 years. We completed preliminary analyses checking for outliers and used VIF to assess for multiple collinearity. We used multiple linear regression to examine the relationship between lifetime marijuana use (independent variable) on each dependent variable. We used separate models for each dependent variable (SCD-self-care score, treating pain at home, being admitted to hospital, and going to the ER/infusion center for pain); and we adjusted each model for gender and education as they are known to be associated with substance use.

## Results

There were 291 participants in our sample. The majority of the sample was greater than or equal to 25 years of age (n = 220) with a minority less than age 25 (n = 71). Among the younger cohort, 16.9% (n = 12/71) reported using marijuana compared to 21.8% (n = 48/220) of the older cohort (Table 1). There were no significant differences in rates of marijuana use by age (p = 0.40). As shown in Table 1, the younger cohort was less likely to have a high school diploma, had fewer days treating pain at home, and had more hospital admissions for pain than the older cohort.

**Table 1:** Baseline characteristics stratified by age in sample of participants with sickle cell disease (SCD).

Characteristic	Total	<25 years of age	≥25 years of age	p-value^a^
		(n = 71)	(n = 220)	
**Lifetime Marijuana Use**	60 (20.6)	12 (16.9)	48 (21.8)	0.40
**Gender,** n (%) Male	134(46.1)	34(47.9)	100(45.5)	0.79
**Education,** n (%) High school diploma or less	185(64.9)	59(84.3)	126(58.6)	<0.01
**Household income**, n (%) <$30,000 annually	127(50.4)	30(55.6)	97(49)	0.44
**Race,** n (%) African American	284(98.3)	70(98.6)	214(98.2)	1.00
**During past month:**^**b**^				
**Days treating pain at home**				
Mean (Median, Range)	1.9(2, 0–4)	1.7(1, 0–4)	2(2, 0–4)	0.03
**Days in ED/infusion center visits for pain**				
Mean (Median, Range)	1.7(2, 0–4)	1.9(2, 0–4)	1.6(2, 0–4)	0.16
**Hospital admissions for pain**				
Mean (Median, Range)	1.4(1, 0–4)	1.9(2, 0–4)	1.2(1, 0–4)	<0.01
**SCD Self-care Scale,** How often do you				
Mean (Median, Range)				
…. drink enough liquids?	3.2(3, 0–4)	3.3(3, 1–4)	3.2(3, 0–4)	0.67
… refrain from over-exercise?	2.6(3, 0–4)	2.6(3, 0–4)	2.6(3, 0–4)	0.60
… keep your clinic appointments?	3.3(4, 1–4)	3.1(3, 1–4)	3.3(4, 1–4)	0.10
… take medications as prescribed?	3.4(4, 0–4)	3.2(3, 0–4)	3.4(4, 0–4)	0.24
… follow the doctor’s instructions?	3.4(3, 1–4)	3.3(3, 1–4)	3.4(3, 1–4)	0.66
(When you see a doctor,) ….make sure that your medical questions are answered?	3.4(4, 0–4)	3.4(4, 2–4)	3.4(4, 0–4)	0.48
**Total Score**	19.2(19, 9–24)	18.9(19, 9–24)	19.3(20, 11–24)	0.40

^a^Comparing the variable values for those endorsing any marijuana use <25 years of age to the variable values for those endorsing any marijuana use > = 25 years of age. We used Fisher’s exact test for the categorical variables and Wilcoxon rank-sum (Mann-Whitney) test for continuous variables.

^b^Individuals were asked (1) how many times during a typical month they treated pain at home with possible response groupings from the original IMPORT study of 0, 1–5, 6–10, 11–20, >20 coded as 0, 1, 2, 3, 4 respectively; (2) how many times they were admitted to a hospital in the last 12 months with categories of 0, 1–2, 3–5, 6–10 or >10 coded as 0, 1, 2, 3, 4 respectively; and (3) how many times in the last 12 months they went to the ER or infusion center for a pain crisis, with response groupings of none, 1–2, 3–5, 6–10, >10 coded as 0, 1, 2, 3, 4 respectively.

When stratifying the analysis by age group and examining differences in sociodemographic and pain/self-care outcomes by marijuana use, we find that the younger age group who reported marijuana use had significantly lower self-care scores compared to youth with no marijuana use (17.1 vs 19.4; p = 0.04). Among the older age group, those who endorsed a lifetime history of marijuana use were more likely to have a high school diploma or less (79.2% vs. 52.1%; p = 0.001), and more likely to have a household income less than $30,000 (69.8% vs. 42.9%; p = 0.002). Those endorsing marijuana use in the older age group also reported increased days treating pain at home (p = 0.01) with no statistically significant differences in other outcomes.

There was evidence of effect modification when stratifying by age. After stratifying the sample by age and adjusting for sex and education, we found the younger cohort reporting a history of marijuana use had lower mean *self-care scores* (β = -2.74 (1.01), p = 0.009; F2.93) compared to those without marijuana use (Table 2). In contrast, there was no significant difference in *self-care scores* (β = -0.27 (0.52), p = 0.606) among the older cohort regardless of marijuana status (Table 2). The younger cohort who reported marijuana use were more likely to have admissions to the hospital for pain compared to those who did not report marijuana use (β = 0.87(0.43),p = 0.0.047). In contrast, among the older cohort who reported regular marijuana use, there were more days when they treated their pain at home (β = 0.44 (0.21), p = 0.035; F = 3.67), but they had had no difference in resulting ED visits (β = 0.23 (0.20), p = 0.252) or hospitalizations (β = -0.01 (0.18), p = 0.968) compared to those who did not use marijuana (Table 2). While the F-statistics are significant for adjusted models examining the relationship of marijuana use on SCD self-care in young adults and days treating pain at home in older adults, the adjusted R-squares are small.

**Table 2:** Relationship between lifetime marijuana use on health-related behaviors and pain management stratified by age.

Variable^a^	<25 years of age^b^		≥25 years of age^c^	
	Beta(SE), P	F, R^2^	Beta(SE), P	F, R^2^
**SCD Self-care**^**d**^				
Unadjusted model	-2.33(0.95), 0.02	6.06, 0.08	-0.5(0.5), 0.32	1.01, 0.01
Adjusted model	-2.74(1.01), 0.01	2.93, 0.08	-0.27(0.52), 0.61	1.45, 0.01
**In past month:**				
**Days treating pain at home**^**3**^				
Unadjusted model	0.2(0.37), 0.59	0.29, 0.004	0.53(0.2), 0.01	7.03, 0.03
Adjusted model	0.23(0.4), 0.57	0.67, -0.02	0.44(0.21), 0.04	3.67, 0.04
**ED/infusion center visits for pain**				
Unadjusted model	0.49(0.4), 0.22	1.51, 0.02	0.3(0.19), 0.11	2.52, 0.01
Adjusted model	0.75(0.43), 0.09	1.26, 0.01	0.23(0.2), 0.25	1.4, 0.01
**Hospital admissions for pain**				
Unadjusted model	0.86(0.4), 0.03	4.66, 0.07	0.08(0.18), 0.63	0.23, 0.001
Adjusted model	0.87(0.43), 0.05	1.57, 0.03	-0.01(0.18), 0.97	1.02, 0.0003

^a^Each dependent variable (SCD self-care, days treating pain at home, in ED/infusion center, and hospital admissions) were adjusted separately for sex and education with lifetime marijuana use as independent variable. For all outcomes, we used linear regression model, reported Beta (SE), F-ratio and R-square for unadjusted model, adjR-square for adjusted model.

^**b**^Number included in analyses from 66–69 based on varied item missingness.

^**c**^Number included in analyses from 208–217 based on varied item missingness.

^**d**^Lower scores are associated with endorsing less self-care behaviors. Sample score range 9–24.

## Discussion

In the IMPORT sample, only a minority of patients with SCD across all ages report a history of marijuana use. While this proportion was lower than that seen in some other studies [5], our sample represents a population of treatment-seeking adolescents and adults with sickle cell disease. The prevalence of lifetime marijuana use was lower than expected based on the prevalence of marijuana use among the general population, suggesting adolescents and adults with sickle cell disease may have less risk for marijuana use, or that patients in our studied may have under-reported their lifetime use. This study demonstrated that different ages with SCD who report marijuana use have different associated self-care and health-related behaviors. The age-related differences may explain some of the discordant findings published on associations of marijuana use and health of patients with SCD. The older cohort who endorsed marijuana use reported more days of treating pain at home, although this did not translate into increased acute care visits for pain crisis. It may be that marijuana use in this older cohort is driven by pain management and marijuana may be used to cope with pain. Although interestingly, despite experiencing more pain at home, the older cohort using marijuana did not report more acute care visits or hospitalization. Our findings are similar to the PISCES study which found experiencing pain at home in adults with SCD does not necessarily lead to increased hospital utilization or acute care visits [14]. The adults in our sample may be less likely to use hospital care to treat their pain for a number of unmeasured variables contributing to confounding, such as, healthcare access, health system trust, previous positive or negative experiences.

In contrast, young adults who used marijuana endorsed lower disease related self-care scores and also more hospital admissions for pain. Ballas (2016) described a similar associated increase in hospitalization for patients using cannabis although they did not stratify by age [5]. While our findings are only associations, our age stratified findings suggest that it could be that marijuana itself does not increase VOC risk, but that marijuana may serve as a surrogate for worse SCD self-care, which itself increases the risk for hospitalization. Alternatively, it may mean that younger people who use marijuana may have worse disease prognosis to begin with and, subsequently, are more likely to be hospitalized–whether they use marijuana or not. Worse disease severity among young people may also contribute to increased pain and more frequent hospitalization leading to both worse self-care behaviors and also an increased use of marijuana for analgesic effect, further confounding the relationship. We need additional prospective trials to definitively tease out both the relationship to pain, self-care, and clinical outcomes.

While our findings are limited as we used observational data that prevents causal inferences, this work provides some key preliminary findings on associations of marijuana use with certain types of health behaviors in sub-populations with SCD. As this was a secondary data analysis, we were limited to analyzing data that was collected for the primary study and lacked information on reasons for marijuana use. Our sample size is limited to an urban population of patients with sickle cell and so cannot be generalized to the general population, although it does provide some insight on a vulnerable subpopulation of patients with serious disease.

It is important than clinicians ask not only about marijuana use in patients with SCD, but also ask about reasons for its use and the impact on pain management. In general, adolescents and young adults exhibit poorer self-care and chronic disease management compared to adults [15] and screening for marijuana use among this age group may help identify those also at elevated risk for lapses in disease management or high utilization of acute care resources. Future research should prospectively analyze factors influencing marijuana use, including reasons for use, in this population and tease out its disease-related health consequences.

## Supporting information

S1 File Audio Computer-Assisted Self-Interview (ACASI) items (Baseline). (PDF)

S2 File (DTA)

## Abbreviation

SCD: Sickle Cell Disease

## References

[1] Ware RE, de Montalembert, Tshilolo L, Aboud MR. Sickle Cell Disease. The Lancet. 2017;390(10091): 311–323. 10.1016/S0140-6736(17)30193-9 28159390

[2] Howard J, Anie KA, Holdcroft A, et al Cannabis use in sickle cell disease: a questionnaire study. Br J Hematol. 2005;131:123–128 10.1111/j.1365-2141.2005.05723.x 16173972

[3] Kalu N, O’Neal PA, Nwokolo C, Diaz S, Owoyemi O. The use of marijuana and hydroxyurea among Sickle Cell Patients. Blood. 2016;128:5913.

[4] Knight-Madden J, Lewis N, Hambleton IR. The prevalence of marijuana smoking in young adults with sickle cell disease: a longitudinal study. West Indian Med J. 2006;55:224–227 10.1590/s0043-31442006000400004 17249310

[5] Ballas SK. The use of cannabis by patients with sickle cell disease increased the frequency of hospitalization due to vaso-occlusive crises. Cannabis and Cannabinoid Research. 2017;2(1):197–201. 10.1089/can.2017.0011 29082316 PMC5627667

[6] Roberts JD, Spodick J, Cole J, Bozzo J, Curtis S, Forray A. Marijuana use in adults living with sickle cell disease. Cannabis and cannabinoid research. 2018;3(1). 10.1089/can.2018.0001 PMC6044416 30014039

[7] Gotlieb VK, OO KZ. Marijuana use and sickle cell disease. Blood. 2008;112:4826.

[8] Winters KC, Arria A. Adolescent brain development and drugs. Prev Res. 2012;18(2): 21–24. PMC3399589 22822298

[9] Davis M, Sheidow A, Zajac K, McCart M. Prevalene and impact of substance use among emerging adults with serious mental health conditions. Psychiatr Rehabil J. 2012; 35(3): 235–243. 10.2975/35.3.2012.235.243 22246122 PMC3767039

[10] Haywood C., Lanzkron S., Carroll C., Strouse J., Bediako S., Haythornthwaite J., et al Perceived discrimiantion in health care is associated with daily chronic pain in sickle cell disease. Blood. 2014; 48(5): 1657–1662. 10.1016/j.jpainsymman.2014.02.002 PMC4198520 24742787

[11] Haywood C Jr, Diener-West M, Strouse J, Carroll CP, Bediako S, Lanzkron S, et al Perceived discrimination in health care is associated with a greater burden of pain in sickle cell disease. J Pain Symptom Manage. 2014; 48(5):934–43. 10.1016/j.jpainsymman.2014.02.002 24742787 PMC4198520

[12] McLellan AT, Cacciola JS, Zanis D. Addiction Severity Index, Lite Version. 1997 [Accessed Nov 11, 2019]; Available from: http://who.int/substance_abuse/research_tools/addictionseverity/en/index.html

[13] Lenoci J, Telfair J, Cecil H, Edwards RR. Development and Testing of a Tool to Assess Self-Care Agency in Adults with Sickle Cell Disease. Western Journal of Nursing Research. 2002;24(3):228–245. 11926191 10.1177/01939450222045879

[14] Smith WR, Penberthy LT, Bovbjerg VE, McClish DK, Roberts JD, et al Daily assessment of pain in adults with sickle cell disease. Ann Intern Med 2008;148: 94–101. 10.7326/0003-4819-148-2-200801150-00004 18195334

[15] Bonnie RJ, Stroud C, Breiner H. Investing in health and well-being of young adults—IOM report. 1^st^ ed Washington, DC: National Academies Press; 2014. 25855847

