# Supplementary material for: Marijuana use and health behaviors in a US clinic sample of patients with sickle cell disease
Source: PLoS One. 2020 Jul 14;15(7):e0235192. doi: 10.1371/journal.pone.0235192 (PMC7360374; doi:10.1371/journal.pone.0235192)
Supplement: S1 File — (PDF) [file pone.0235192.s001.pdf]

Audio Computer-Assisted Self-Interview (ACASI) Items

(Baseline)

Trust and Experiences of Respect

Variable	Trust in Medical Professionals ; 0 to 100 scale; higher scores = more trust
Source	Dugan E, Trachtenberg F, Hall M. Development of abbreviated measures to assess patient trust in a physician, a health insurer, and the medical profession. BMC Health Services Research. 2005;5:64.
Response Options	Strongly Disagree – Strongly Agree
Items	We would like to ask you a few questions about how you feel about doctors in general. Please indicate your level of agreement with the following statements using the scale (insert response options)<br><br>1. Sometimes doctors care more about what is convenient for them than about their patient's medical needs<br>2. Doctors are extremely thorough and careful<br>3. You completely trust doctors' decisions about which treatments are best<br>4. A doctor would never mislead you about anything<br>5. All in all, you trust doctors completely
Variable Name	r_dugantrust1 dugantrust1-dugantrust5 dugantrustscore
**Variable**	**Healthcare System Distrust ; 10 to 50 scale; higher scores = more distrust**
Source	Rose A et al. J Gen Intern Med 2004;19(1):57-63.
Response Options	Strongly agree---strongly disagree (5 points) (R = reverse coded)
Items	Please tell me how you feel about the following statements. Do you: strongly agree, agree, disagree, strongly disagree, or are you not sure?<br>1. Medical experiments can be done on me without my knowing it.<br>2. My medical records are kept private.(R)<br>3. People die every day because mistakes are made in the health care system.<br>4. When they take my blood, they do tests they don't tell me about.<br>5. If a mistake were made in my health care, the health care system would try to hide it from me.<br>6. People can get access to my medical records without my approval.<br>7. The health care system cares more about holding costs down that it does about what is needed for my health.<br>8. I receive high-quality medical care from the health care system.(R)<br>9. The health care system puts my medical needs above all other considerations when treating my medical problems.(R)<br>10. Some medicines have things in them that they don't tell you about.
Variable Name	rosedistrust1 – rosedistrust10 r_rosedistrust2 r_rosedistrust8 r_rosedistrust9 rosedistrustscore
**Variable**	**Experiences of Respect/Provider Communication ; 0 to 100 scale; higher scores = more positive ratings of provider communication**
Source	Consumer Assessment of Health Plans Survey (CAHPS)
Response Options	Never – Always (4 points)
Items	Now I would like you to think about your experiences with doctors and other health providers over the last 12 months. This includes all of your experiences over the past year, not just limited to this visit.<br><br>1. In the last 12 months, how often did doctors or other health providers listen carefully to you?<br>2. In the last 12 months, how often did doctors or other health providers explain things in a way you could understand?<br>3. In the last 12 months, how often did doctors or other health providers show respect for what you had to say?

	2. How difficult is it get care or the medical advice you need from your sickle cell doctor in the evenings (very difficult, somewhat difficult, not too difficult, or not at all difficult)<br>3. And, thinking about your visits to your sickle cell doctor, how often do you find the visits well organized and running on time? Would you say always, often, sometimes, rarely, or never?<br>4.Do you have a regular doctor, besides your sickle cell provider, who you usually go to when you are sick or need health care? (yes, know, don't know, refused)<br><br>Based on those who have a regular source of care…<br>*How difficult is it to contact your doctor or a medical person at your regular place of care over the telephone about a health problem? (very difficult, somewhat difficulty, not too difficulty, not at all difficult, don't know, refused)<br>*How difficult is it get care or the medical advice you need in the evenings (very difficult, somewhat difficult, not too difficult, or not at all difficult)<br>*And, thinking about your visits to your regular doctor or regular place for care, how often do you find the visits well organized and running on time? Would you say always, often, sometimes, rarely, or never?<br>3. In the last 2 years, have you visited an emergency room? (yes, know, don't know, refused)<br>4. In the last 2 years, have you been admitted to the hospital? (yes, know, don't know, refused)<br>5. Where do you USUALLY go when you are sick OR need health care? (doctor's office or private clinic, community health center or other public clinic, hospital outpatient department, hospital emergency room, some other place, don't know, refused)<br>6. The last time you were sick or needed medical attention in the past 2 years, how quickly could you get an appointment to see a doctor or health professional? Did you get an appointment (on the same day, the next day, in 2-3 days, in 4-5 days, in 6-7 days, after more than a week, never able to get appointment, not sick nor needed urgent care in past 2 years, went to ER/Urgent Care where appointment isn't needed)?
**Variable Name**	pcmh1 – pcmh11
**Variable**	**Sickle Cell Self-Care Behaviors; Scored on 0 to 24 scale, with higher scores = more frequent self-care practices**
**Source**	Adapted from Lenoci et al.
**Response Options**	Never, Seldom, Sometimes, Often, Very Often
**Items**	The following questions ask you to think about how often you do certain things related to your sickle cell disease. There are no right or wrong answers…<br><br>1) How often do you drink enough liquids?<br>2) How often do you refrain from over-exercise?<br>3) How often do you keep your clinic appointments?<br>4) How often do you take medications as prescribed?<br>5) How often do you follow the doctor's instructions?<br>6) When you see a doctor, how often do you make sure that your medical questions are answered?
**Notes**	The six responses are added, forming a composite score, with higher scores representing more frequent self-care practices
**Variable Name**	scdselfcare1 - scdselfcare6 scdselfcarescore
**Variable**	**Sickle Cell Self Efficacy Scale (SCSES); Scored on 9 to 45 scale, with higher scores = greater perceived sickle cell self-efficacy**
**Source**	Edwards et al.
**Response Options**	Not at all sure, Not sure, Neither, Sure, Very Sure
**Items**	1) How sure are you that you can do something to cut down on most of the pain you have when having a pain episode?<br>2) How sure are you that you can keep doing most of the things you do day-to-day?<br>3) How sure are you that you can keep sickle cell disease pain from interfering with your sleep?<br>4) How sure are you that you can reduce your sickle cell disease pain by using methods other than taking extra medication?

	**scores = more sleep difficulties; isi_totalcat is scored as follows: 0 to 7(no insomnia); 8 to 14(subthreshold insomnia); 15 to 21(moderate insomnia); 22 to 28(severe insomnia)**
**Source**	Insomnia Severity Index
**Response Options**	Varies
**Items**	1a. * How severe is your current difficulty falling asleep?<br>1b. * How severe is your current difficulty staying asleep?<br>1c. * How severe is your current problem waking up too early?<br>2. * How satisfied or dissatisfied are you with your current sleep pattern?<br>3. * To what extent do you consider your sleep problem to interfere with your daily functioning?<br>4. * How noticeable to others do you think your sleeping problem is in terms of impairing the quality of your life?<br>5. * How worried/distressed are you about your current sleep problem?
**Variable Name**	sleep1a sleep1b sleep1c sleep2 sleep3 sleep4 sleep5 isi_total isi_totalcat
**Variable**	**Depressive Symptoms; Scored on 0 to 30 scale, higher scores = more depressive symptoms**
**Source**	Center for Epidemiologic Studies Depression (CES-D) Scale
**Response Options**	Rarely/None of the time – Most/All of the time (4 points)
**Items**	Below is a list of some of the ways you may have felt or behaved. Please indicate how often you have felt this way during the past week:<br>-Rarely or none of the time (less than 1 day)<br>-Some or a little of the time (1-2 days)<br>-Occasionally or a moderate amount of time (3-4 days)<br>-Most or all of the time (5-7 days)<br>1. I was bothered by things that usually don't bother me.<br>2. I had trouble keeping my mind on what I was doing.<br>3. I felt depressed.<br>4. I felt that everything I did was an effort.<br>5. I felt hopeful about the future.<br>6. I felt fearful.<br>7. My sleep was restless.<br>8. I was happy.<br>9. I felt lonely.<br>10. I could not "get going."
**Variable Name**	cesd1 - cesd10 r_cesd5 r_cesd8 cesd10score
**Variable**	**Other health related questions**
**Source**	
**Response Options**	yes, no, don't know
**Items**	1. Are you currently being treated for depression? (yes, no, don't know)<br>2. Have you ever been treated for depression? (yes, no, don't know)<br>3. In general, how would you describe your own health? (excellent, very good, good, only fair, poor, don't know)
**Variable Name**	depress1 depress2 generalhealth

**Variable**	**History of Substance Use and Abuse**
**Source**	McLellan AT, Cacciola JS, Zanis D. Addiction Severity Index, Lite Version (ASI-Lite). 1997 [Accessed on 14 Feb. 2007]; Available from: URL:http://www.who.int/substance_abuse/research_tools/addictionseverity/en/index.html
**Response Options**	
**Items**	I'm going to ask you some questions about your alcohol and other drug use and any problems you may have had in these areas. I would like to remind you that the information you give me is confidential, and will only be used for research purposes. For the following questions, the time frames will be for two different periods; for the past 30 days, and in your lifetime. For lifetime use, I am interested in the number of years that you used 3 or more times per week.

	For each item, ask:<br>How many days in the past 30 days have you used (name of drug) ?<br>How many years in your life have your regularly used (name of drug) ? Regular use means 3 or more times per week, binges, problematic irregular used in which normal activities are compromised.<br>For all except alcohol ask:<br>How have you most commonly used (name of drug) in the last 30 days (oral, nasal, smoking, non IV injection, IV injection, or never used)?<br>For methadone, other opiates/analgesics, amphetamines, marijuana/cannabis, also ask:<br>Was it prescribed?<br>Was it used without a prescription?
**Variable Name**	drugs_methdays drugs_methyears drugs_methroute drugs_methrx drugs_methnorx drugs_methabuse drugs_othopsdays drugs_othopsyears drugs_othopsroute drugs_othopsrx drugs_othopsnorx drugs_othopsabuse drugs_alcohdays drugs_alcohyears drugs_alcohintoxdays drugs_alcohintoxyears drugs_alcohintoxabuse drugs_heroindays drugs_heroinyears drugs_heroinroute drugs_heroinabuse drugs_cokedays drugs_cokeyears drugs_cokeroute drugs_cokeabuse drugs_amphetdays drugs_amphetyears drugs_amphetroute drugs_amphetrx drugs_amphetnorx drugs_amphetabuse drugs_weeddays drugs_weedyears drugs_weedroute drugs_weedrx drugs_weednorx drugs_weedabuse

		**Past 30 Days (# of days)**	**Lifetime (# of years)**	**Route of administration**<br>**1 = oral**<br>**2 = nasal**<br>**3 = smoking**<br>**4 = non-IV-injection**<br>**5 = IV injection**	**Prescribed?**	**Used without prescription?**
**1**	Alcohol (any use at all)	__ __	__ __			
**2**	Alcohol (to intoxication)	__ __	__ __			
**3**	Heroin	__ __	__ __	1 2 3 4 5		
**4**	Methadone	__ __	__ __	1 2 3 4 5	☐ (1)	☐ (1)
**5**	Other opiates/analgesics	__ __	__ __	1 2 3 4 5	☐ (1)	☐ (1)
**6**	Cocaine	__ __	__ __	1 2 3 4 5		
**7**	Amphetamines	__ __	__ __	1 2 3 4 5	☐ (1)	☐ (1)
**8**	Marijuana/Cannabis	__ __	__ __	1 2 3 4 5	☐ (1)	☐ (1)

Personal, Social and Behavioral Characteristics

**Variable**	**Life Stress/Instability; Scored on 0 to 84 scale, with higher scores = more life stress**
**Source**	Harrell, S.P. (1994). The Urban Life Stress Scale (ULSS). Unpublished instrument.
**Response Options**	4 = Extreme stress, more than I feel I can handle

	10. People lose jobs when employers learn they have sickle cell.<br>11. I worry about people discriminating against me because I have sickle cell.
**Variable Name**	scdstigma1 - scdstigma11 stigma_social stigma_internal stigma_disclose stigma_discrim
**Variable**	**Demographics**
**Response Options**	Varies
**Items**	1. What do you consider to be your main racial or ethnic group? Choose only one. (White/Caucasian, Black/African American, Hispanic/Latino, American Indian, Asian, Pacific Islander/Native Hawaiian, Other (please specify))<br>2. Where you born in the United States? (yes/no)<br><br>3. What is your sex? (Male, Female)<br>4. What is your primary language spoken at home? (English, Spanish, French, Other (please specify))<br>5. Where do you live now? (homeless (living on the street, in a park, in a bus station, etc.), in a shelter, single-room occupancy hotel, staying with friends/family, rent an apartment/house , own my home, other (please specify))<br>6. Number of people living in the home age 17 and older.<br>7. Number of people living in the home age 16 or younger.<br>8. Now, think about your yearly family income for the household. By household income, I mean your income, plus the income of all the members living in your household, including cohabiting partners.<br>a) <$10,000<br>b) $10,000-$19,999<br>c) $ 20,000-29,999<br>d) $30,000-39,999<br>e) $40,000-49,999<br>f) $50,000-59,999<br>g) $60,000-69,999<br>h) $70,000-79,999<br>i) $80,000-89,999<br>j) $90,000-99,999<br>k) $100,000-$150,000<br>l) >$150,000<br>m) refused to answer<br>n) unknown<br><br>9. What is the highest degree or diploma you have? (None/less than high school, High school diploma or GED, Associate degree/junior or 2-year college, Bachelors or 4 year college degree, Graduate or professional degree)<br>10. Are you currently in school? (Yes/No)<br>11. At this time, are you: (Working full time or part time, With a job and on sick leave, With a job and not working for other reasons, Laid off, Unemployed and looking for work, Not working or not looking for work, Disabled and not working, Or retired and not working, None of the above)?<br>12. What type of insurance do you currently have? (Medicare, Medicaid, Private, Self-Pay, Don't Know)
**Variable Name**	study_id jhusite howardsite age adult18 adult21 female race2 blackrace raceo bornus primlang prilango currentresidence livenowo adultsinhome kidsinhome peoplehouse employment employment2 income2 incomecat poverty income_median school education education2 ins_medicare ins_medicaid ins_private ins_selfpay ins_ssd ins_mhip ins_other inso insurance
